# Adipose tissue derived stromal cells in a gelatin-based 3D matrix with exclusive ascorbic acid signalling emerged as a novel neural tissue engineering construct: an innovative prototype for soft tissue

**DOI:** 10.1093/rb/rbac031

**Published:** 2022-05-24

**Authors:** Catherine Ann Martin, Subathra Radhakrishnan, Jose Luis Gómez Ribelles, Omana Anna Trentz, Nivethaa EAK, Mettu Srinivas Reddy, Mohamed Rela, Narayana Kalkura Subbaraya

**Affiliations:** Crystal Growth Centre, Anna University, Guindy, Chennai-600025, India; Cell Laboratory, National Foundation for Liver Research, No.7, CLC works Road, Chennai-600044, India; Cell Laboratory, National Foundation for Liver Research, No.7, CLC works Road, Chennai-600044, India; Center for Biomaterials and Tissue Engineering (CBIT), Universitat Politècnica de València, Valencia 46022, Spain; Biomedical Research Networking Centre in Bioengineering, Biomaterials and Nanomedicine (CIBER-BBN), Valencia 46022, Spain; MIOT Institute of Research, MIOT Hospitals, 4/112, Mount Poonamallee Road, Chennai-600089, India; Crystal Growth Centre, Anna University, Guindy, Chennai-600025, India; Cell Laboratory, National Foundation for Liver Research, No.7, CLC works Road, Chennai-600044, India; Cell Laboratory, National Foundation for Liver Research, No.7, CLC works Road, Chennai-600044, India; Crystal Growth Centre, Anna University, Guindy, Chennai-600025, India

**Keywords:** adipose derived stem cells (ASCs), ascorbic acid, soft tissue engineering, triad, neuronal differentiation

## Abstract

The current study investigated a triad, which comprises of adipose tissue derived stem cells isolated from infrapatellar fat pad and gelatin/polyvinyl alcohol (PVA)-based matrix with exclusive ascorbic acid signalling. Though, the bio-mechanical properties of the gelatin–PVA blended scaffolds in wet condition are equivalent to the ECM of soft tissues in general, in this study, the triad was tested as a model for neural tissue engineering. Apart from being cytocompatible and biocompatible, the porosity of the scaffold has been designed in such a manner that it facilitates the cell signalling and enables the exchange of nutrients and gases. The highly proliferative stem cells from Passage 2 were characterized using both, mesenchymal and embryonic stem cell markers. As an initial exploration the mesenchymal stem cells at Passage 4 were exposed to ascorbic acid and basic fibroblast growth factor signalling for neuronal differentiation in 2D environment independently. The MSCs successfully differentiated and acquired neuron specific markers related to cytoskeleton and synapses. Subsequently, three phases of experiments have been conducted on the 3D gelatin/PVA matrix to prove their efficacy, the growth of stem cells, growth of differentiated neurons and the *in situ* growth and differentiation of MSCs. The scaffold was conducive and directed MSCs to neuronal lineage under specific signalling. Overall, this organotypic model triad could open a new avenue in the field of soft tissue engineering as a simple and effective tissue construct.

## Graphical Abstract

**Figure:**
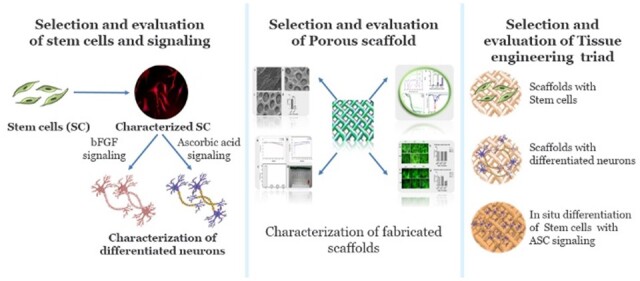

## Introduction

Clinical studies in India have reported that ∼72% of the trauma cases resulted in huge soft tissue loss [1, 2]. Soft tissue damage also occurs due to ageing, disease, tumours, congenital defects and acquired deformities [3]. The replacement of the damaged soft tissue by autologous flap reconstruction is considered as one of the efficient therapeutic approaches [4]. But the limitations like donor site morbidity and volume loss have established a pressing clinical need for better restoration of soft tissues. Clinicians and researchers worldwide have investigated numerous biomaterials made up of either synthetic or natural polymers for soft tissue engineering. The scaffolds can be used as either an implant, only as a supporting material without cells or it can be used as an organotypic 3D construct with active components such as growth/differentiation factors and proliferating cells to facilitate functional tissue regeneration.

Soft tissue engineering aims at the regeneration/restoration of tissues like brain, fat, tendons, ligaments, muscles, fibrous tissues and blood vessels. The fibre reinforced composite ECM of soft tissues is mainly composed of collagen, elastin and ground substance (gelatinous amorphous material). Various probing techniques have revealed that the mechanical strength of soft tissues ranges from 0.1 kPa to 1 MPa [5]. The heterotypic fibrils of Collagen I and Collagen III present in the microenvironment of soft tissues were found to affect the mechanical properties by regulating fibril dimensions [6]. In addition, the hydrated proteoglycans such as glycosaminoglycans (GAGs) endows resistance against compressive forces in soft tissues [7]. The primary objective of this study is to fabricate a representative model for soft tissue engineering which would replace the lost soft tissue by recapitulating the developmental mechanisms.

In the present study, the properties such as porosity, topography, water uptake, degradation, biocompatibility and cytocompatibility of gelatin–polyvinyl alcohol (PVA) blended scaffolds were studied in a controlled manner. The amorphous gelatin (natural polymer) and PVA (synthetic polymer) are blended in such a way that it mimics the mechanical properties of native ECM of soft tissues. Additionally, the resistance of scaffolds against the degradative property of the body fluids in vivo allows this matrix to provide a long-term support to the biological activity of cells that aids in tissue regeneration [8]. Hydration of the scaffolds is considered as the characteristic feature of soft tissue constructs as it supports the diffusion of nutrients and cell dynamics. In contrast to cellular therapy, such organotypic 3D matrix provides the niche-like support to the cells which facilitates tissue restoration.

The resected infrapatellar fat pad (IFP), during knee arthroplasty is a medical/surgical waste. In this study, the IFP/Hoffa pad served as the rich source of human adipose tissue derived stem cells (ASCs). The mesenchymal stem cells that reside in IFP have multilineage differentiation potential and good proliferation kinetics [9, 10]. Besides, these cells also have the unique ability to secrete the ECM proteins of soft tissues such as Collagen I and Collagen III [11]. A varied array of cytokines like Vascular endothelial growth factor (VEGF), platelet derived growth factor (PDGF), basic fibroblast growth factor (bFGF) and transforming growth factor β (TGF-β) are also secreted by the ASCs which provides the signals for angiogenesis, anti-apoptosis and proliferation [12]. Such marked advantages in the functional regulation, proliferation and differentiation of ASCs make them an ideal candidate for large scale studies in translational medicine.

Last but not the least, the presence of essential signalling factors is mandatory to guide the specific differentiation of ASCs seeded in the scaffold. l-Ascorbic acid (AA), a potent radical scavenger is found to be involved in tissue homeostasis. Recently, extensive studies have been carried out in the involvement of AA in stem cell biology from normal proliferation that aids in *in vitro* expansion to epigenome reprogramming during differentiation [13, 14]. The functional recovery of injured peripheral nerves, restoration of spinal cord injury, neuroepithelial cell maintenance and yield increase in neurons revealed the influential regulation of AA signalling in dopaminergic neuron development [15–18]. This report analysed the efficacy of AA as an exclusive signalling molecule in the differentiation of ASCs towards neural lineage cells. Our previous studies reported that ASCs have the inherent ability to differentiate into neural lineage cells in later passages > P6 [19]. We hypothesise that the cells at Passage 4 of ASCs as transit amplifying cells towards neural lineage. These cells were exposed to AA and bFGF (positive control) signalling independently for further differentiation in 2D environment. In addition to test the efficacy of the scaffold, the *in situ* neuronal differentiation was performed with the stemness rich Passage 2 stem cells. The organotypic model supports the growth and proliferation of stem cells and in the presence of AA signalling, it enhances the neuronal differentiation. Thus, the novel representative neural tissue engineering triad encompasses gelatin–PVA blended scaffolds, ASCs from IFP and AA signalling.

## Materials and methods

### Materials

The materials used in this study comprise polymers and other chemicals which include Gelatin Type B (Merck), PVA (MW—40 000) (Merck) and Glutaraldehyde (Sigma) of 99% purity for the fabrication of the biomaterial, using double distilled water. The chemicals for cell culture include Dulbecco’s Modified Eagle’s Medium (DMEM) (Gibco), Dulbecco’s Phosphate Buffered Saline (DPBS) cell culture grade, antibiotic–antimycotic (Invitrogen), foetal bovine serum (FBS) (Invitrogen), bFGF (Invitrogen), paraformaldehyde (sigma), Triton X-100 (sigma) and Bovine serum albumin (Sigma). All the reactions were carried using Millipore grade water for cell culture.

### Human IFP collection from patients undergoing knee arthroplasty

Ethical approvals were obtained from The Institutional Ethics Committee (IEC) and the Institutional Committee for Stem Cell Research (IC-SCR) of Gleneagles Global Health City, Chennai, for the collection of IFP after a written consent from the patients. All procedures were carried out in accordance with the National Guidelines for Stem Cell Research-2017, India.

### Isolation and expansion of human ASCs

The isolation of ASCs was carried out using the previously described method [20]. The collection of adipose samples in sterile phosphate buffered saline (PBS) was transported to the stem cell laboratory within 30 min with sterile handling. DPBS (Lonza) was used for washing of fat tissue before it was minced into small pieces, in a 50-ml tube (Amicon). This was digested overnight with 6 ml of 0.075% collagenase type I (PAN Biotech) in DPBS, in a CO_2_ incubator at 37°C. DMEM (Gibco) with 10% FBS was added to the collagenase digested tissue, 24 h after incubation. This diluted tissue digest was filtered through a 70-µm cell strainer (BD Biosciences). This single cell suspension was centrifuged at 1800 rpm for 8 min, and the pellet was washed with DPBS several times before plating onto a tissue culture plate (TCP) (58 cm^2^) (Cellstar^®^ Greiner Bio-one, Germany) in DMEM with 10% foetal calf serum and 5% of an antibiotic–antimycotic mixture (Gibco) (Complete Medium). These plates were incubated at 37°C, 95% humidity, and 5% CO_2_. Fresh medium was added to the adherent cells after 48 h, after washing off of the non-adherent cells with DPBS. The medium was replaced twice a week and once the cells reached confluency about 80–85% they were trypsinized and sub-cultured.

### Morphological analysis

After the cells reached 80% confluency, they were closely monitored for morphological changes using an inverted phase contrast microscope (Nikon Ts2FL, Japan). A confluent layer of ASCs was fixed using 4% paraformaldehyde and then stained with Mayer’s haematoxylin using the standard protocol. The cells were then mounted using di-*n*-butylphthalate in xylene solution [21].

### Immunocytochemistry

Immunocytochemistry (ICC) is a technique for the detection and visualization of proteins in their particular localization in single cells. Cells from Passage 2 Day 14 for ASCs and Passage 5 Day 45 for neural lineage cells, were cultured on a cover slip in 35 mm petri dish, washed with DPBS and fixed using 4% paraformaldehyde at 4°C overnight. The ICC protocol was performed as previously described [19]. The antibodies used in the study are tabulated in Table 1. Total corrected fluorescence of the cells were calculated using the formula and analysed using Image J software. Statistical analyses were performed on biological triplicates obtained from three different patients (*n* = 3).

$$\text{Corrected total cell fluorescence}\;\left(\text{CTCF}\right)=\text{Integrated density}-(\text{area of selected cell}\times \text{mean fluorescence of background})$$

**Table I.** Primary and secondary antibodies

Protein	Manufacturer	Catalogue number
Neuron specific enolase (NSE)	Santa Cruz Biotechnology, Inc.	sc-51880
Neurofilament light (NF-L)	Santa Cruz Biotechnology, Inc.	sc-20012
Synaptosome associated protein 25 (SNAP25)	Santa Cruz Biotechnology, Inc.	sc-7539
Syntabulin	Santa Cruz Biotechnology, Inc.	sc-87447
Glyceraldehyde-3-phosphate dehydrogenese) GAPDH	Santa Cruz Biotechnology, Inc.	sc-47724
CD166	Santa Cruz Biotechnology, Inc.	sc-8549
Endoglin CD105	Santa Cruz Biotechnology, Inc.	sc-18838
Nanog	Santa Cruz Biotechnology, Inc.	sc-30331
Nucleostemin	Santa Cruz Biotechnology, Inc.	sc-46218
Syntrophin	Biolegend	845102
GAP 43	Santa Cruz Biotechnology, Inc.	sc-7458
MAP 2	Santa Cruz Biotechnology, Inc.	sc-74421
Dopamine Receptor D4	Biolegend	850101
OLIG 2	Santa Cruz Biotechnology, Inc.	sc-19967
ASCL 1	Santa Cruz Biotechnology, Inc.	sc-13219
GDNF	Santa Cruz Biotechnology, Inc.	sc-13147
Secondary antibodies		
m-IgGkBP-FITC	Santa Cruz Biotechnology, Inc.	sc-516140
Mouse anti-goat IgG—FITC	Santa Cruz Biotechnology, Inc.	sc-2356
Mouse anti-goat IgG—HRP	Santa Cruz Biotechnology, Inc.	sc-2354
m-IgGkBP-HRP	Santa Cruz Biotechnology, Inc.	sc-516102
Mouse anti-goat IgG—PE	Santa Cruz Biotechnology, Inc.	sc-3452
m-IgGkBP-PE	Santa Cruz Biotechnology, Inc.	sc-516141

### Reverse transcriptase-polymerase chain reaction

RNA was isolated from ASCs and neuronal cells (appropriate passages) using a RNeasy Mini Kit (Qiagen, cat. no. 74104). The purity of the RNA was confirmed using the 260/280 nm absorbance ratio, and the RNA was quantified using a Bio Photometer D30 (Eppendorf). The isolated RNA was stored at −80°C until use. For reverse transcription, 1 µg of total RNA was reverse transcribed using a First Strand cDNA Synthesis Kit (Thermo Scientific, K1622) according to the manufacturer’s protocol. Each reaction was prepared with a total volume of 20 µl and carried out at 37°C for 60 min in a Master Cycler Pro-S (Eppendorf). The resulting cDNA was considered optimally pure and used for reverse transcriptase-polymerase chain reaction (RT-PCR). PCR amplification was performed on 3 µl (<200 ng) of cDNA using HotStarTaq plus Master Mix (Qiagen) with Coral Load Concentrate (Qiagen, cat. no. 203443) Each PCR reaction was prepared with a total volume of 25 µl. The thermal cycler (Master Cycler Pro-S (Eppendorf)) was programmed according to the manufacturer’s instructions. After amplification, the samples were stored at 4°C in the short term or at −30°C to –15°C for longer storage. PCR products were confirmed by agarose gel electrophoresis. The gel was then visualized under a transilluminator (Vilber Lourmat). Primers are listed in Table 2.

**Table 2.** Oligonucleotide sequence used in RT-PCR

Gene	Primer sequence
DCX	For.: 5′-AATCCCAACTGGTCTGTCAAC-3′
	Rev.: 5′-GTTTCCCTTCATGACTCGGCA-3′
NFM	For.: 5′-TGGGAAATGGCTCGTCATTT-3′
	Rev.: 5′-CTTCATGGAAGCGGCCAATT-3′
NF-L	For.: 5′-TCCTACTACACCAGCCATGT-3′
	Rev: 5′-TCCCCAGCACCTTCAACTTT-3′
β-Tubulin III	For.: 5′-AGTGATGAGCATGGCATCGA-3′
	Rev.: 5′-AGGCAGTCGCAGTTTTCACA-3′
NURR1	For.: 5′-ATCTCCTGACCGGCT CTATG-3′
	Rev.: 5′-TGGGTTGGACCTGTATGCTA-3′
EN1	For.: 5′- TGGGTGTACTGCACACGTTATTC-3′
	Rev.: 5′-GGAACTCCGCCTTGAGTCTCT-3′
GAPDH	For.: 5′-CTCGTGGAGTCTACTGGTGT-3′
	Rev.: 5′-GTCATCATACTTGGCAGGTT-3′
CD105	For.: 5′-TGTCTCACTTCATGCCTCAGCT
	Rev.: 5′-AGGCTGTCCATGTTGAGGAGT
Nucleostemin	For.: 5′-GGGAAGATAACCAAGCGTGTG
	Rev.: 5′-CCTCCAAGAAGTTTCCAAAGG
CD166	For.: 5′-AGATACCATTATCATCATACCTTGCCGACT
	Rev.: 5′-TGTCTTTGTATTCGTGTACATCGTCG
CD44	For.: 5′-GAT CCA CCC CAA TTC CAT CTG TGC-3′
	Rev.: 5′-AAC CGC GAG AAT CAA AGC CAA GGC C-3′
β−actin	For.: 5′-CCCAGCACAATGAAGATCAA-3′
	Rev.: 5′-ACATCTGCTGGAAGGTGGAC-3′
CD13	For.: 5′-GAAGGCCATGTTCAACATCACAC-3′
	Rev.: 5′-GATTCCAATCTGGACACCATTGG-3′

### Neural induction

#### Neural induction using bFGF

The Passage 4/5 ASCs kept in complete medium was replaced with serum-free DMEM medium with 20 ng/ml of bFGF (known neuronal inducer) and 5% PAN serum replacement. The cells were maintained for 7 days in the same medium with periodical change of fresh medium for every 3 days. Later the bFGF containing medium was removed and washed with DPBS. Serum-free medium with 5% PAN serum replacement (Panexin NTA, PAN Biotech catalogue no. PO4-95700) was added to the cells. This temporal exposure (mimics *in vivo* exposure) of bFGF aids neuronal induction. The morphological changes were observed under Nikon Ts2FL phase contrast microscopy [22].


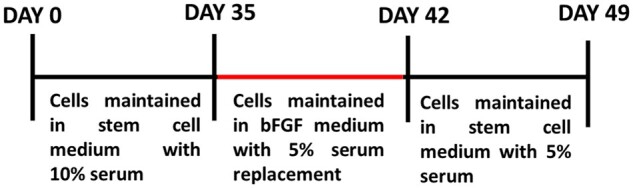


#### Neural induction using AA

The Passage 4/5 ASCs kept in complete medium was replaced with serum-free DMEM medium with 200 µM of AA an antioxidant (Sigma) and 5% PAN serum replacement. The cells were maintained for 30 days in the same medium with periodical change of fresh medium for every three days. The exposure of AA aids in neuronal induction. The morphological changes were observed under Nikon Ts2FL phase contrast microscopy.


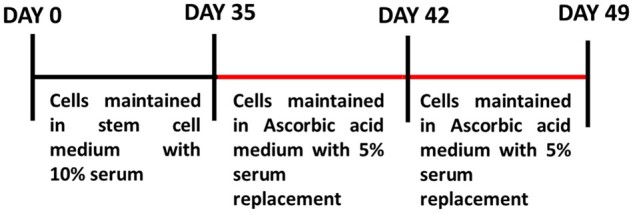


### Scaffold fabrication

Gelatin and PVA were dissolved in water. PVA and gelatin were taken such that the weight ratio was 7.5:20 and 7.5:40 (PVA concentration kept constant). The solutions were mixed with constant stirring and poured in a polypropylene petri dish Tarsons, (diameter: 9.2 cm, thickness: 1.2 cm) was allowed to gelate overnight at 25°C, which was followed by incubation at −20°C for 12 h. The scaffold after incubation was lyophilized at −110°C and pressure of −0.002 mbar for 6 h [23]. The samples containing PVA and gelatin (PVA: gelatin) in different ratios 7.5:20 and 7.5:40 will be referred to as PVA20 and PVA40, respectively. The scaffolds fabricated were cross-linked using 10 ml of 0.4% glutaraldehyde (Sigma) overnight and rinsed using deionized water to remove the traces of glutaraldehyde present in the scaffold. The absence of Glutaraldehyde was confirmed using FT-IR. The scaffolds were pre frozen at (−20°C for 12 h) and lyophilized as mentioned afore.

### Material characterization

#### Surface topology and porosity measurements

The shape and morphology of prepared scaffolds and the cell–material interaction were studied using scanning electron microscopy (SEM, Carl Zeiss MA15/EVO 18) at an accelerated voltage of 15 keV. The scaffold was coated with gold using a sputter coater (Quorum). The pore size measurements were carried out for the obtained SEM micrographs using Image J software (National Institute of Health, Bethesda, MD, USA). Porosity measurement of the scaffolds was carried out using solvent replacement method. The dried scaffolds were immersed in ethanol overnight and then excess ethanol on the surface was dried before weighing. The porosity of the scaffolds was calculated using the formula

$$\text{Porosity}=\left(M_{2}-{\;M}_{1}\right)/\rho V\times 100$$

where, M_1_ and M_2_ are the mass of the scaffold before and after immersion in ethanol, respectively, ρ is the density of absolute ethanol and *V* is the volume of the scaffold [24, 25].

#### Physical characterization using thermal and mechanical analysis

The thermal degradation of the gelatin–PVA scaffolds, PVA20 and PVA40 using the thermogravimetric method in a Mettler Toledo, Star 2 system Columbus, OH, at a steady scanning rate of 10°C/min under oxygen atmosphere. This was carried out on 5 mg samples in the temperature range of 25–900°C. The tensile strength (TS) of the scaffolds was carried out following the ASTM guidelines using universal testing machine (UTM) at room temperature (UTM, H10KS, Tinius Olsen, UK using the standard of ASTM D638). The samples with dimensions 1 mm × 9 mm × 155 mm was used for the analysis, with a gauge length of 25 mm at a cross head speed of 10 mm/min. TS of the scaffolds was expressed in MegaPascal.

#### Biophysical characterization using FT-IR, swelling, degradation and hemocompatibility

The structural information of the parental compound gelatin, PVA and the scaffolds with their various concentrations cross-linked with glutaraldehyde was sought using Attenuated Total Reflection FT-IR (ATR-FT-IR). The IR spectra was obtained using Jasco International Co./Japan Fourier Transform Infrared Spectrometer Model FTIR-6300, over a range of 400–4000 cm^−1^ at a resolution of 4 cm^−1^. A conventional technique was used to assess the capacity of swelling and for confirmation of the crosslinking reactions. The experiments were carried out in DMEM (cell culture medium), as previously described. Hemocompatibility is an important feature of any implantable biomaterial, and this was carried out in accordance with previous protocols [26]. *In vitro* biodegradability was carried out at 37°C in lysozyme solution. The freeze-dried scaffolds were cut into circles of 1 cm diameter and a weight of 10 mg. The concentration of the lysozyme solution taken was 0.8 µg/ml in PBS which is approximately half of the concentration present in human serum. The samples were incubated in enzyme solution for predetermined time intervals. The samples were removed at regular intervals washed with deionized water for the removal of salts and then freeze dried. The weight after enzymatic degradation was recorded [27]. The % of lysozyme degradation was determined by the percentage of weight loss using the formula:

$\%\;\mathrm{Degradation}\;=\;\left(\frac{W_{0}-\;W_{1}}{W_{0}}\right)\times \;100$(2)

where $W_{0}\;$denotes the initial weight of the samples; $W_{1}$ denotes the final weight after enzymatic action.

### Cell material interaction using MTT and live/dead assay

The ASCs and neuronal proliferation rate on the porous scaffolds and TCP were evaluated on Day 1, 3 and 5 for ASCs and Day 5, 7 and 11 for differentiated neuronal cells, using the MTT assay. The distribution and viability of ASCs and differentiated neurons seeded on the scaffold were studied using acridine orange (AO)/propidium iodide (PI) staining. These assays were carried out according to previously described methods [26].

### *In situ* differentiation of stem cells on the gelatin/PVA matrix

The cells from Passage 2 at Day 16 was seeded on the scaffolds and allowed to differentiate in AA medium for a period of 21 days after which the gene expression of the neuronal markers was studied using semi quantitative RT-PCR. The string analysis of the protein–protein interaction of the genes responsible for mesencephalic dopaminergic neurons have been analysed.

## Results

### Characterization of Passage 2 stem cells isolated from Hoffa pad/IFP revealed their mesenchymal origin with immense self-renewability

**Figure 1.**
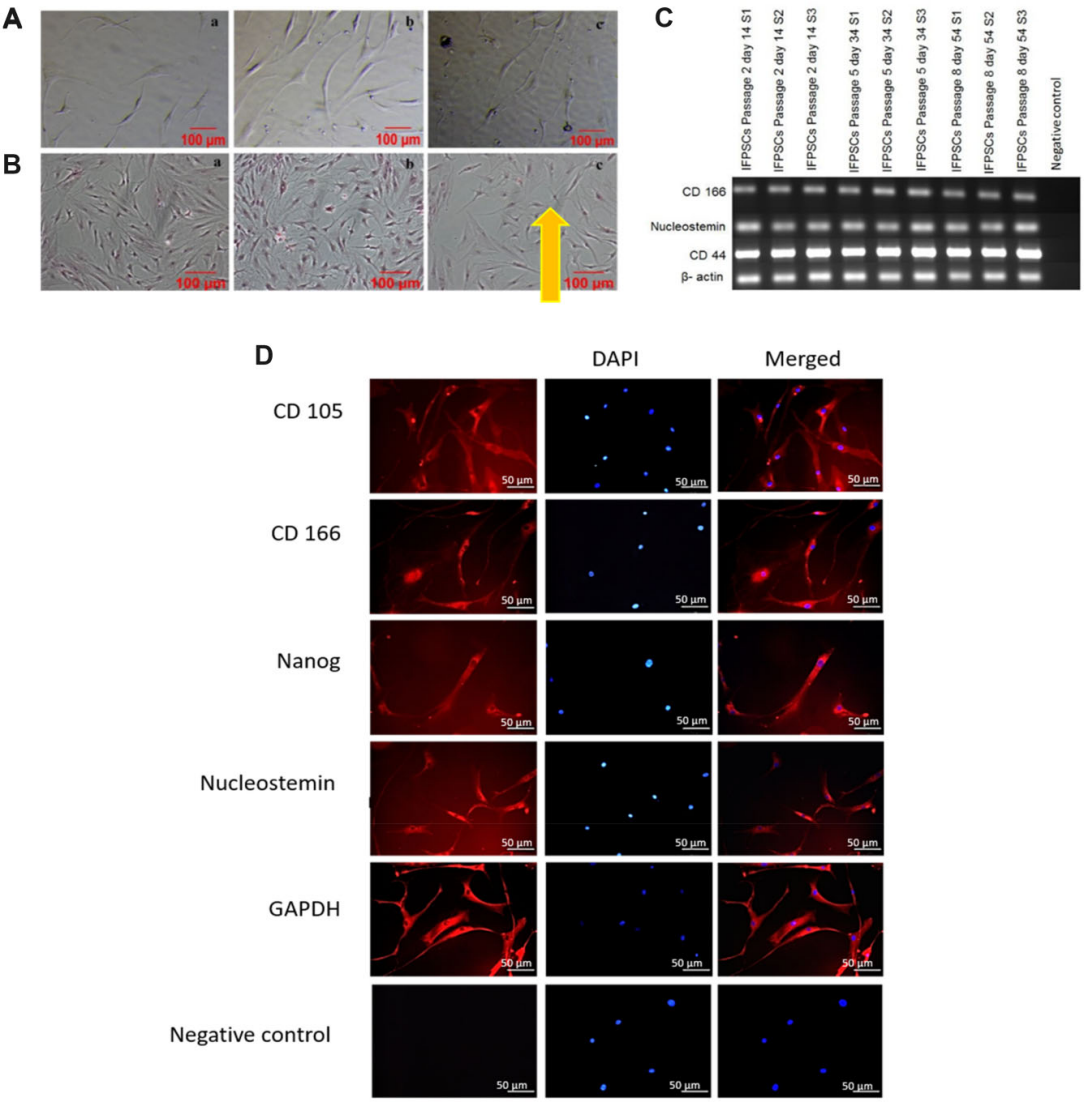
Morphology and characterization of isolated ASCs. Morphological analysis of ASCs using phase contrast microscopy (**A**). Haematoxylin and eosin staining of ASCs (**B**). Cells from all the three passages were thin, displayed fibroblast structures, and possessed distinct nuclei. ASCs exhibited tapering ends and short projections. A small fraction of cells from Passage 8—Day 54 showed stress fibres; these cells were relatively broad. Gene expression was characterized by semi-quantitative RT-PCR. Two mesenchymal markers CD44 and CD166 were constitutively expressed until Passage 8. The proliferative marker nucleostemin was also present persistently in Passages 2–8. β-Actin served as the endogenous control (**C**). Representative immunofluorescence images of ASCs stained with specific primary antibodies and secondary antibodies tagged with PE (**D**). CD105, CD166, nanog, nucleostemin, GAPDH an endogenous positive control and unstained cells. The expression of the markers in the isolated ASCs show they have ESC and MSC properties.

ASCs were successfully isolated from human IFP tissue obtained from patients undergoing knee arthroplasty surgery. These ASCs were cultured in DMEM complete medium, and they displayed healthy spindle shaped morphology. These cells were passaged for every 7 days. These cells have good proliferation ability and could be passaged healthily for 8 times, since the cells displayed stress fibres (Fig. 1A, yellow arrow) at later passage and population doubling time also significantly decreased. So, the early passage cells P2 and P3 were used for characterization. The expression of marker genes in the ASCs was determined by reverse transcription-PCR. It was revealed that the ASCs not only expressed the mesenchymal stem cell marker genes as CD44 and CD166, but also expressed stromal proliferation marker nucleostemin where β-actin serves as endogenous control (Fig. 1B). The isolated ASCs from Passage 2 were analysed with specific antibodies to determine the localization and expression of cell surface markers (CD105 and CD166) and stem cell markers (nanog and nucleostemin). The analysis indicated that the ASCs highly expressed all the markers tested (Fig. 1C). These markers expressed by the stem cells are Cell adhesion molecules (CD 166), signalling molecules (CD105), receptors for ECM (CD44) and transcription factor (nanog) which precisely involved in cell–cell and cell–matrix interaction. Altogether, our results suggest that the isolated ASCs are unspecialized cells with self-renewing property.

### Characterization of transdifferentiated neurons derived from ASCs using the exclusive signalling factor, AA proves the differential potential of ASCs

**Figure 2.**
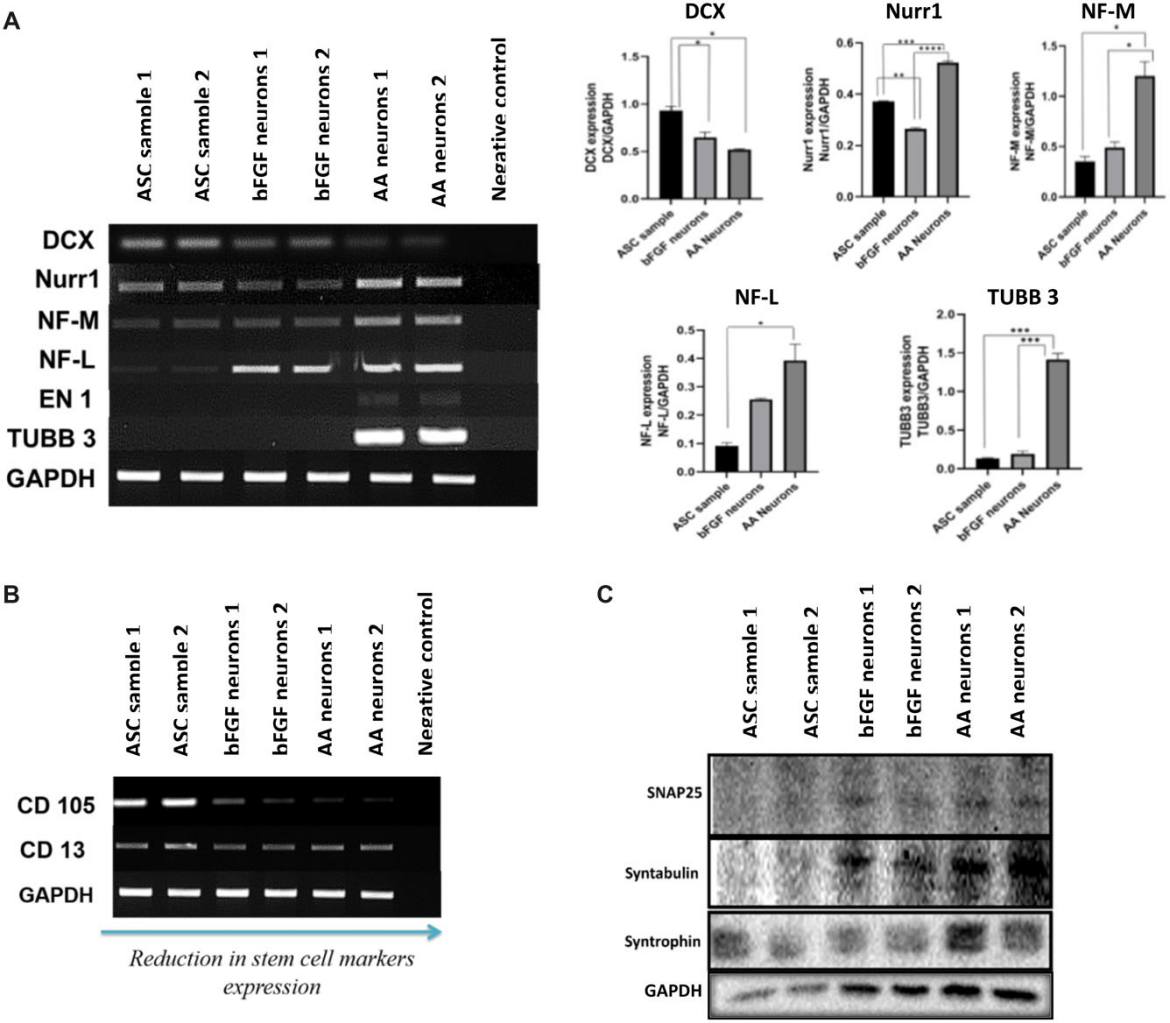
Gene expression studies during the differentiation protocol of ASCS to neuronal cells using bFGF and AA as independent external signalling molecules. RT-PCR analysis of differentiated neural lineage cells (**A** and **B**). the neural specific markers NF-L, NF-M, TUBB-3, EN-1 and NURR-1 are expressed in the differentiated neurons. The early neuronal marker DCX was found to be decreased in the differentiated neurons (A). the mesenchymal markers CD105 and CD13 have been reduced after differentiation (B). GAPDH served as the endogenous control. Quantification was conducted by image J software and the results were normalized to GAPDH. Statistical analysis was conducted using one-way analysis of variance with post hoc Tukey test. The values are expressed as the mean ± SEM. *P < 0.05, **P < 0.01, ***P < 0.001, ****P < 0.0001. Immunoblotting analysis of synaptic protein expression changes during neuronal differentiation of ASCs (**C**).

Our previous studies suggest that stem cells derived from IFP secretes neurotrophins and spontaneously differentiates into neurons at later passages [19]. In this study to test the differentiation potential of the stem cells, P4 ASCs were taken for neural induction. The bFGF (positive control as bFGF is known neural inducer) and AA (antioxidant and dopamine neuron inducer) were used as independent single factors for neural induction. The AA induced differentiation of ASCs resulted in neural lineage cells with significantly increased expression of NF-L, NF-M, TUBB3 and NURR-1 comparatively with bFGF induced neurons (Fig. 2A). Undifferentiated ASC served as the control. Doublecortin (DCX), a protein associated with microtubule involved in neuronal migration present in neural precursor cells in cortical structures [28]. Hence it is considered as an early neuronal marker was significantly reduced in both the differentiated neurons which revealed the later stages of differentiation in both groups. Nurr1 plays a key role in the maintenance of the dopaminergic system of the brain [29]. There was a significant rise in the expression of Nurr1 in AA differentiated neurons. The intermediate filament NF-L (68 kDa) is an integral constituents of the neuron involved in neuronal plasticity and neuronal architecture [30]. NF-L was significantly increased in both differentiated neuronal cells when compared with the control. Conversely, NF-M (125 kDa) another filament which forms dimers with NF-L was 2-fold highly expressed in AA neurons when compared with the other groups. TUBB3, beta tubulins which forms heterodimer with microtubules involved in axon guidance and maintenance, considered as a specific neuronal marker. There was a threefold increase in the expression of TUBB3 in AA differentiated neurons when compared with the control and bFGF differentiated neurons (Fig. 2A). Concomitantly, the TBR 1 transcription factor which is present in post mitotic neurons was completely absent (data not shown). The expression of the stem cell markers CD105 and CD13 were found to diminish in both the groups (Fig. 2B). Altogether these results revealed that the neurons are well differentiated with good proliferative capacities.

The expression of the differentially expressed neuronal -specific proteins in the two approaches during neuronal differentiation was carried out by western blot analysis (Fig. 2C). The differentiation of ASCs was performed by bFGF and AA and at Day 21, after induction the cells showed a mature neuronal morphology. At this stage, the differentiating cells in both groups indicate positivity for intermediate filament, NF-L, Synapsis associated proteins, SNAP-25, Syntabulin and Syntrophin, and a mild positive for axonal protein, GAP-43, indicating the progressing protein synthesis. The absence of multipotent precursor/progenitor markers ASCL-1 and OLIG-2 show that the cells are moving towards maturation and have crossed the precursor stage. The mild presence of certain proteins in ASCs is in accordance with our previous studies indicating ASCs can move towards neuronal lineage in long term culture.

**Figure 3.**
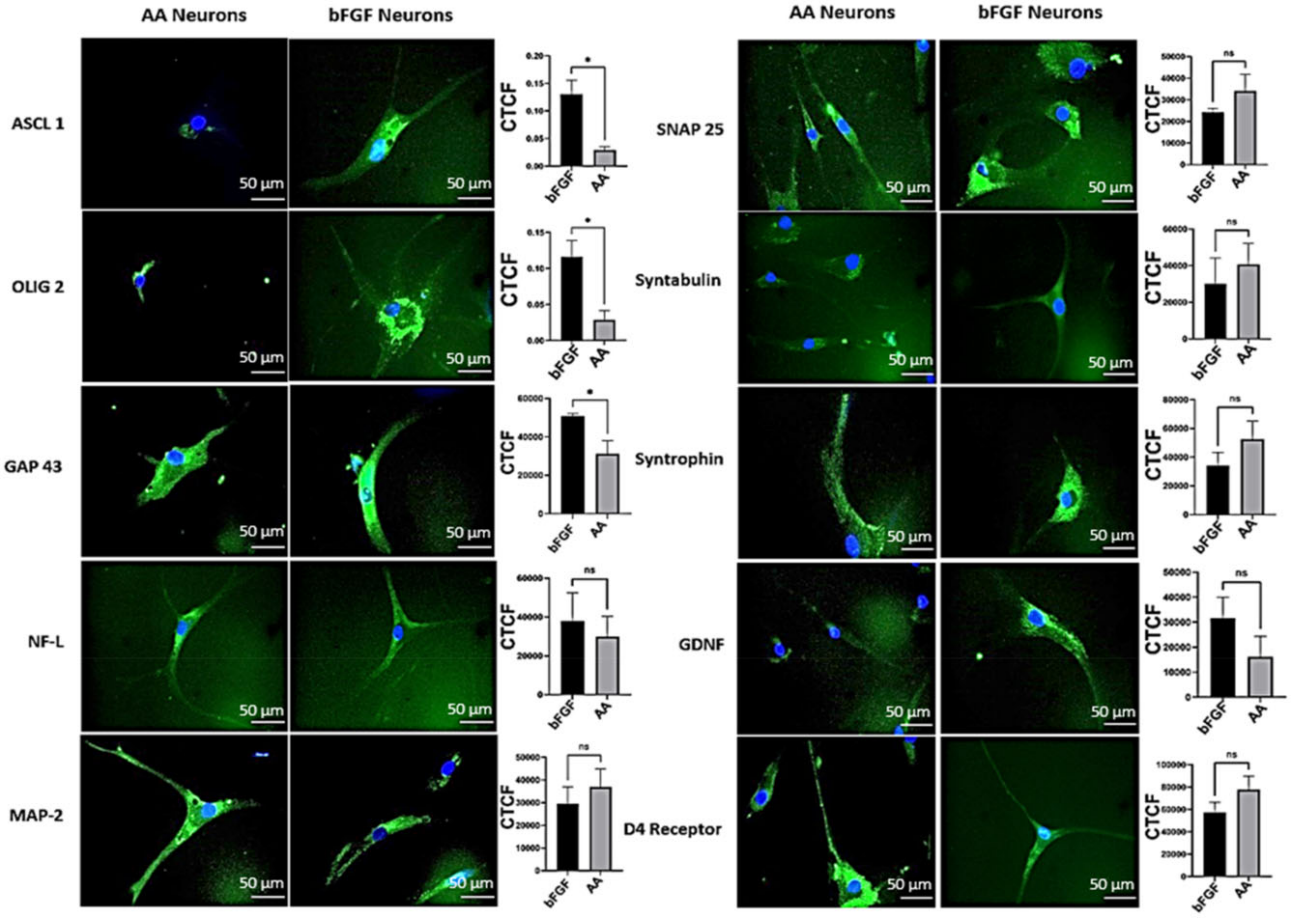
Immunocytochemical analysis of AA differentiated neural lineage cells in comparison to bFGF differentiated neural lineage cells. ASCL-1 (i), OLIG-2 (ii), GAP-43 (iii), NF-L (iv), MAP-2 (v), SNAP-25 (vi), syntabulin (viii), syntrophin (viii), GDNF (ix), D4 receptor (x). endogenous control GAPDH. Corrected total cell fluorescence (CTCF) was calculated (arbitrary units) using image J software and graph plotted using graph pad prism. Statistical analysis was carried out using unpaired T test. The values are expressed as the mean ± SEM. *P < 0.05.

Furthermore, we did a comparative analysis of localization of neuron-specific proteins using immunostaining techniques. The corrected total cell fluorescence was calculated using image j analysis and the graph was plotted using prism (Fig. 3). The basic helix-loop-helix (bHLH) transcription factors ASCL1 and OLIG2 were found to be significantly increased in bFGF differentiated neurons than AA differentiated neurons. ASCL1, is a proneural transcription factor involved in neuronal cell lineage commitment and differentiation and is also critical to the generation of neuroendocrine cells [31]. Similarly, OLIG2 is a multipotent precursor marker, the olig2+ cells can differentiate into either cholinergic neuron in the basal forebrain or oligodendrocytes depending on external signals [32]. The reduction in early neuronal markers in AA-derived neurons indicates that they are later differentiation state. Indeed, the matured synaptic proteins SNAP25, syntabulin and syntrophins and D4 dopamine receptor were found be expressed higher in AA-derived neurons. The cytoskeleton proteins NF-L, NF-M, MAP2 specific to neuronal lineage cells were found to be expressed in both groups. The GDNF is highly expressed in bFGF derived neurons as bFGF aids in establishment of glial-lineage cell [33]. In total, our observations clearly indicates that AA-derived neurons are well developed than bFGF-derived neurons and have well defined cytoskeletal structures with functional synaptic and receptor proteins.

### Physical and biological characterization of fabricated gelatin–PVA blended scaffold revealed that they are hemocompatible and meant for soft tissue engineering which facilitate nutrient/gas exchange

#### The surface morphology of freeze-dried 3D matrix comprising varied ratio of gelatin to PVA

**Figure 4.**
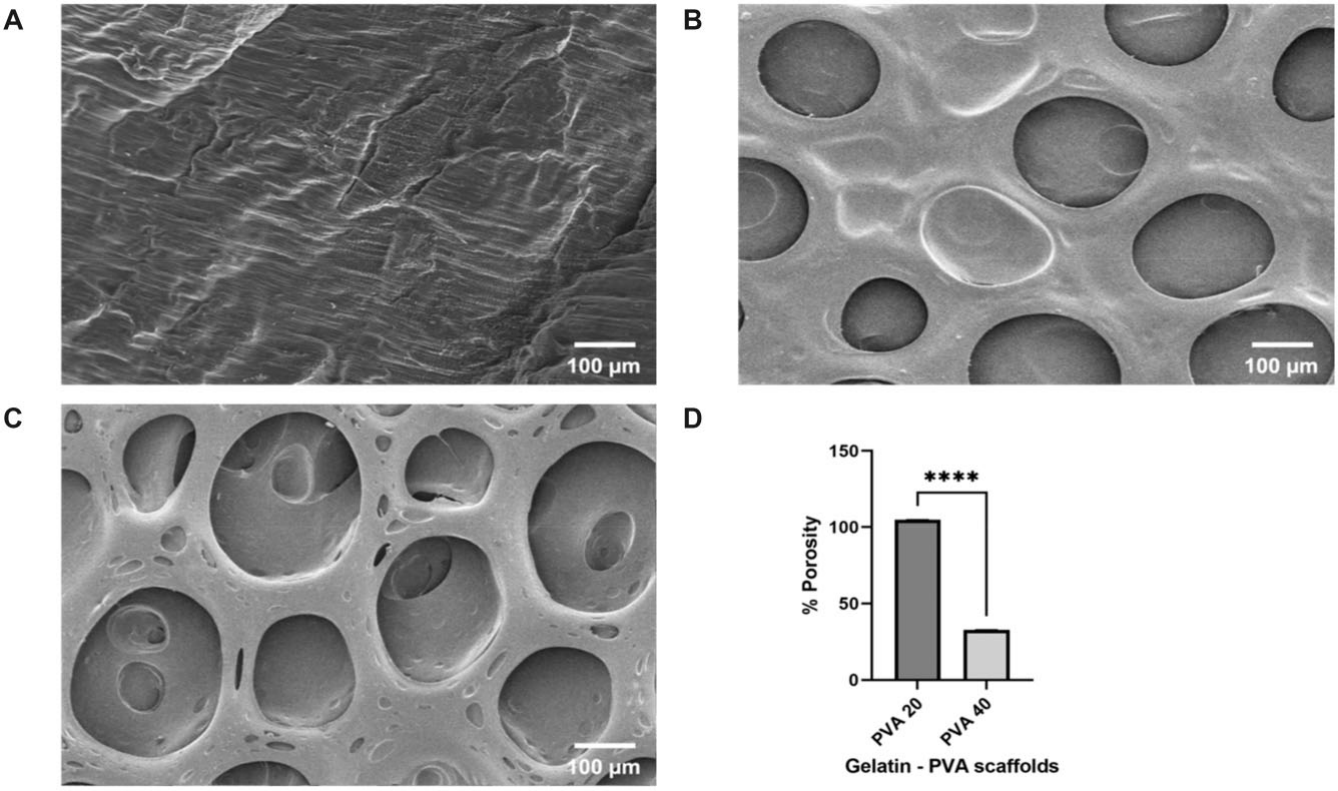
Surface morphological analysis of gelatin–PVA blended scaffolds. SEM micrographs of pure PVA (**A**), PVA20 (**B**), PVA40 (**C**) scaffolds showing an interpenetrating porous structure. The percentage of porosity was calculated using solvent replacement method (**D**). The graph was plotted using graph pad prism. Statistical analysis was carried out using unpaired *T*-test. The values are expressed as the mean ± SEM. ****P < 0.0001.

In general, the protein concentration (natural polymer), agitation speed affects the porosity and mechanical properties of the scaffold [34]. Accordingly, the surface morphology and size of pores was studied using SEM and the percentage of porosity was determined using solvent replacement method. The plain PVA scaffolds were seen to be non-porous with grooves, thus portraying a rough morphology, whereas the PVA20 scaffolds have uniform elongated pores with size ranging from 105 to 115 µm thus forming an inter-penetrating network (IPN). The PVA40 scaffolds have larger irregular pores forming a heterogeneous porous structure ranging from 110–150 µm (Fig. 4A–C). There is a significant increase in the porosity of PVA 20 (*P* < 0.0001) rather than PVA 40 (Fig. 4D). It is clearly evident that gelatin has its direct effect on forming IPN structures. Such polymer blended scaffolds with open pores support the cell dynamics through the interconnected porous matrix and facilitate the exchange of gases and nutrients [27].

#### The FT-IR spectrum of the blend scaffolds shows the polymeric association

**Figure 5.**
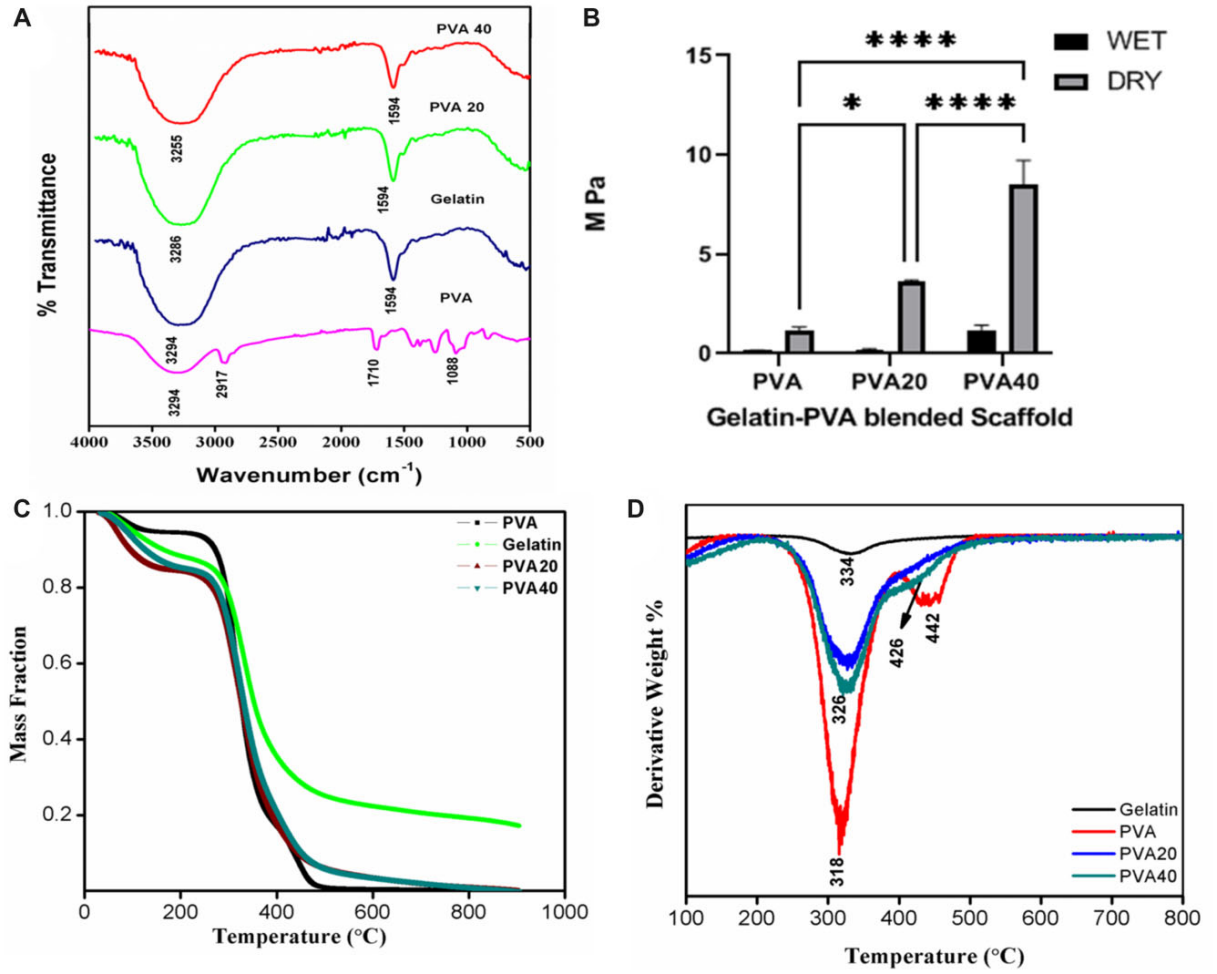
Physiochemical characterization of gelatin–PVA blended scaffolds. FT-IR spectrum of gelatin, PVA 40, PVA 20 and PVA (**A**). TS of gelatin–PVA scaffolds in dry and wet states (**B**). The graph was plotted using graph pad prism. Statistical analysis was carried out using one-way analysis of variance with post hoc Tukey test. The values are expressed as the mean ± SEM. *P < 0.05, *P* < 0.0001. Thermogravimetric analysis of PVA, gelatin, PVA20 and PVA40 (**C**). derivative thermo gravimetric analysis of the PVA/gelatin scaffolds (**D**).

The FT-IR spectra of parental compounds such as PVA, gelatin, along with their composites PVA20 and PVA40 is shown in Fig. 5A. The peak at 3294 cm^−1^ corresponds to the hydrogen bonds (O–H) of PVA and N-H stretching of gelatin. The signature peak at 1710 cm^−1^ of PVA is attributed to C=O and C–O stretch from the acetate group. The characteristic peak of gelatin at 1594 cm^−1^ corresponds to the carboxylate anion C=O stretching vibration. The peak at 2917 cm^−1^ of PVA is attributed to C–H stretching (alkyl group). The absence of prominent peaks between 1260 and 1400 cm^−1^, indicates that it is not Type A gelatin. The crosslinking reaction between PVA and gelatin could be observed by the colour change from white to yellow due to the aldimine linkage CH=N. The broad peak and the lower wavenumber that appears at 3255–3286 cm^−1^ in the composites indicates polymeric association through hydrogen bonding (3150 cm^−1^). In addition, the peak at 1594 cm^−1^ in the parental compounds was observed in the gelatin–PVA composites, which also confirms the polymerization. The non-appearance of the peaks at 1711 cm^−1^ confirms the absence of glutaraldehyde in the crosslinked composite scaffolds [35, 36].

#### Mechanical analysis

Biomechanics of polymeric materials play an essential role in the *invivo* or clinical scenario. Mechanical properties of the scaffolds directly have an impact on cellular processes such as cell proliferation and growth. It has been reported that the mechanical force is transmitted to the innermost part of the cell, through dynamic regulation of cytoskeleton integrity and tension [7, 37]. The mechanical properties pertaining to the TS of the samples in dry and wet state were studied using a stress–strain universal testing equipment. The bio-fabricated scaffold, with varied concentrations of gelatin exhibited a TS ranging from 0.2 MPa (PVA20 in wet condition) to 1.66 MPa (PVA40 in wet condition) that correspond to the mechanical properties of soft tissues. With the addition of gelatin the porosity of the scaffolds decreased linearly which enhances the structural integrity, that is reflected in the TS. The scaffolds in the wet state have a much lower mechanical stiffness than in the dry state. Glutaraldehyde (crosslinker) concentration has a direct impact on the TS of the polymer scaffolds. Mechanical strength improves gradually on increasing the glutaraldehyde up to a certain extent and excess glutaraldehyde leads to brittleness. All the scaffolds exhibit high elasticity which is very favourable for tissue engineering. The results are shown in Fig. 5B.

#### Thermal analysis

The thermograms obtained on this system show three stages of weight loss, the first, from room temperature to 200°C is ascribed to water evaporation, and the two thermal degradation processes appear as shown in Fig. 5C for PVA40 and PVA20. To allow a comparison of the thermograms of the different samples, the weight was normalized to that at 200°C, i.e. the degradation of the dry samples is compared, and first derivative is normalized accordingly. Figure 5D shows the thermograms shifted in the vertical axis to obtain a clearer representation. The shift is by 20% from curve to curve in the case of weight loss and 0.5% K^−^^1^ in the case of the first derivative. PVA presents two degradation processes with first derivative maxima at 319 and 447°C, respectively. On the other hand, gelatin shows a single, broader degradation process with peak maximum at 330°C. In the blends, the low-temperature peak of PVA falls in the same temperature interval than the peak of gelatin and only a small shoulder in the low-temperature side of the peak in the first derivative could be observed. The high-temperature process of PVA can be observed in both blends. Interestingly, the residual weight above 600°C is only 4.4% in PVA but it is quite significant in pure gelatin 22.4%. The residual weight in the blends is 22.0% in PVA20 and 18.0% in PVA40. The derivative plot is shown in Fig. 5D where the interaction of the component in the scaffold is seen.

#### Lysozyme degradation

**Figure 6.**
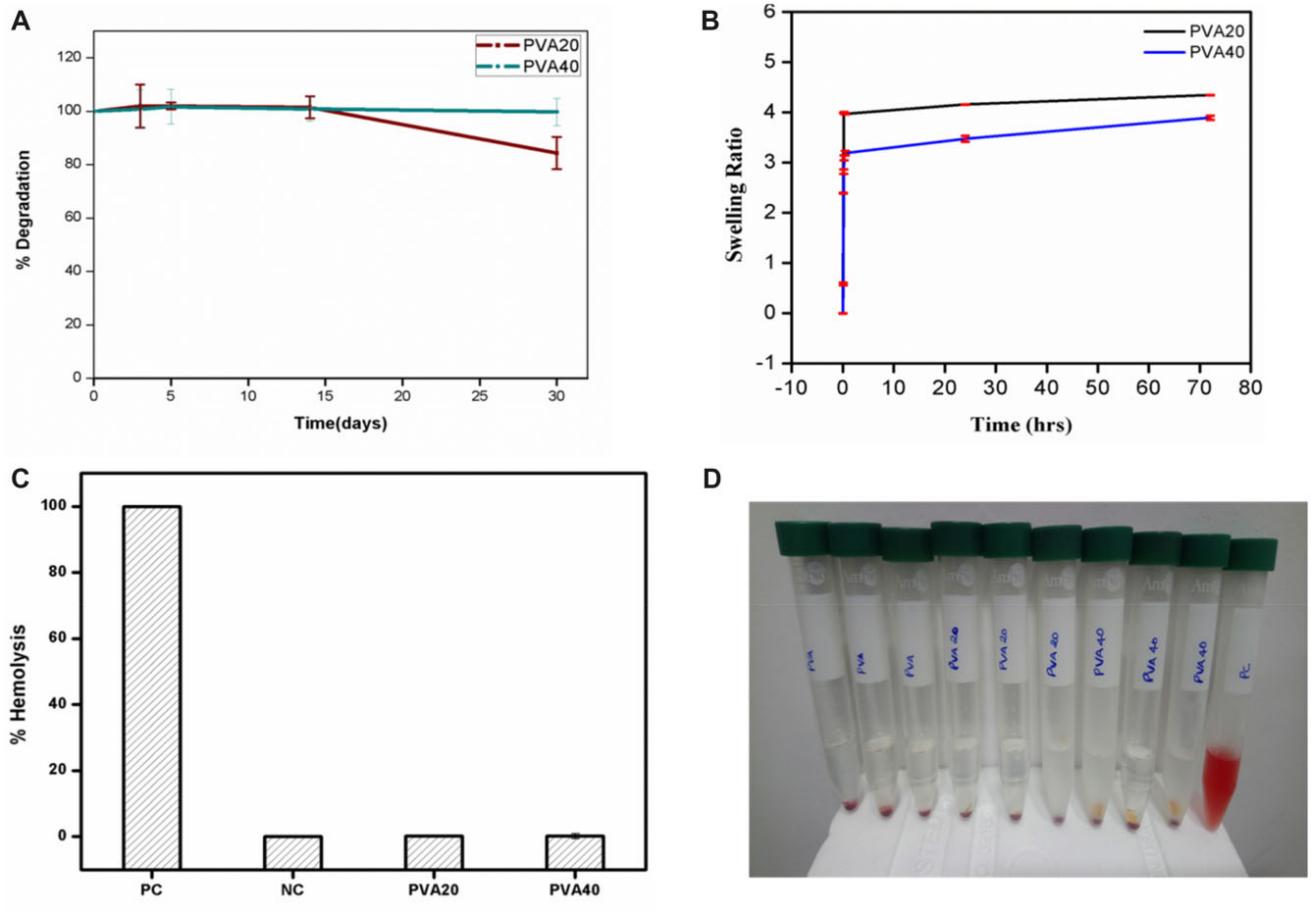
Biological characterization of gelatin–PVA blended scaffolds. *In vitro* degradation of the scaffolds in lysozyme enzyme over a period of 30 days. (**A**) Swelling ratio of the scaffolds at predetermined time intervals. (**B**) Hemocompatibility of PVA/gelatin scaffolds (**C** and **D**), where PC and NC represent positive control and negative control, respectively.

Lysozyme degradation reveals the effect of lysozyme/muramidase (effector of phagocytic cells) on the scaffolds over a period of 30 days. The weight loss of the scaffolds depends on the choice of polymer, the concentration of the polymers, the crosslinker used and the degree of crosslinking [27]. In the blend of gelatin–PVA, gelatin is the naturally occurring degradable polymer. The degradation of gelatin through lysozyme takes place due to the hydrolytic cleavage which affects the overall disintegration of the scaffold. Figure 6A shows the degradation pattern of the PVA/gelatin blended composite scaffolds. The presence of PVA in the composite slows down the degradation. PVA is resistant to hydrolytic cleavage but must dissolve or break down due to physical erosion [38]. The degree of degradation is only 1% in PVA40 and 15% in PVA20 over a period of 30 days.

#### Swelling

Swelling is an inherent feature of biomaterials or hydrogels as it allows the distribution of nutrients and cellular metabolites due to enlargement of the void space between the polymeric networks. The higher the swelling ratio higher the rate of diffusion of nutrients and removal of cellular wastes out of the matrix. The rate of swelling depends on various external factors such as pH, temperature, ionic strength and intrinsic factors such as porosity, pore size, distribution of the pores and choice and concentration of the crosslinker used during synthesis [39, 40]. Figure 6B shows the swelling ratio of the fabricated scaffolds. It is evident from the graph that once the equilibrium is reached within a few hours the scaffolds are stable up to a period of 30 days. Although the water absorbing capability of PVA20 is higher than PVA40 both the composites are highly intact in cell culture medium at 37°C. All the scaffolds stabilize after 24 h and are highly supportive in cell culture media all through the experiments. The high and stable swelling of the scaffolds show that they are suitable for tissue engineering and drug delivery applications.

#### Hemocompatibility

Hemocompatibility is considered one of the major criteria for the *in vivo* applicability of blood contacting materials. The *invitro* analyses according to ISO 10993-4 must be carried out before *invivo* testing [41]. The scaffolds were subjected to static haemolytic model, where the blood cell attachment, thrombus formation and fibrin networks were analysed. The percentage haemolysis is shown in Fig. 6C, and the non-destruction of the blood components is visualized in Fig. 6D. It is evident that the scaffolds are highly hemocompatible, where the haemolysis of the scaffolds is lesser than 1%.

### The fabricated 3D matrix with a blend of gelatin and PVA have been proven to be good for soft tissue engineering as it supports the growth of stem cells and differentiated neurons

**Figure 7.**
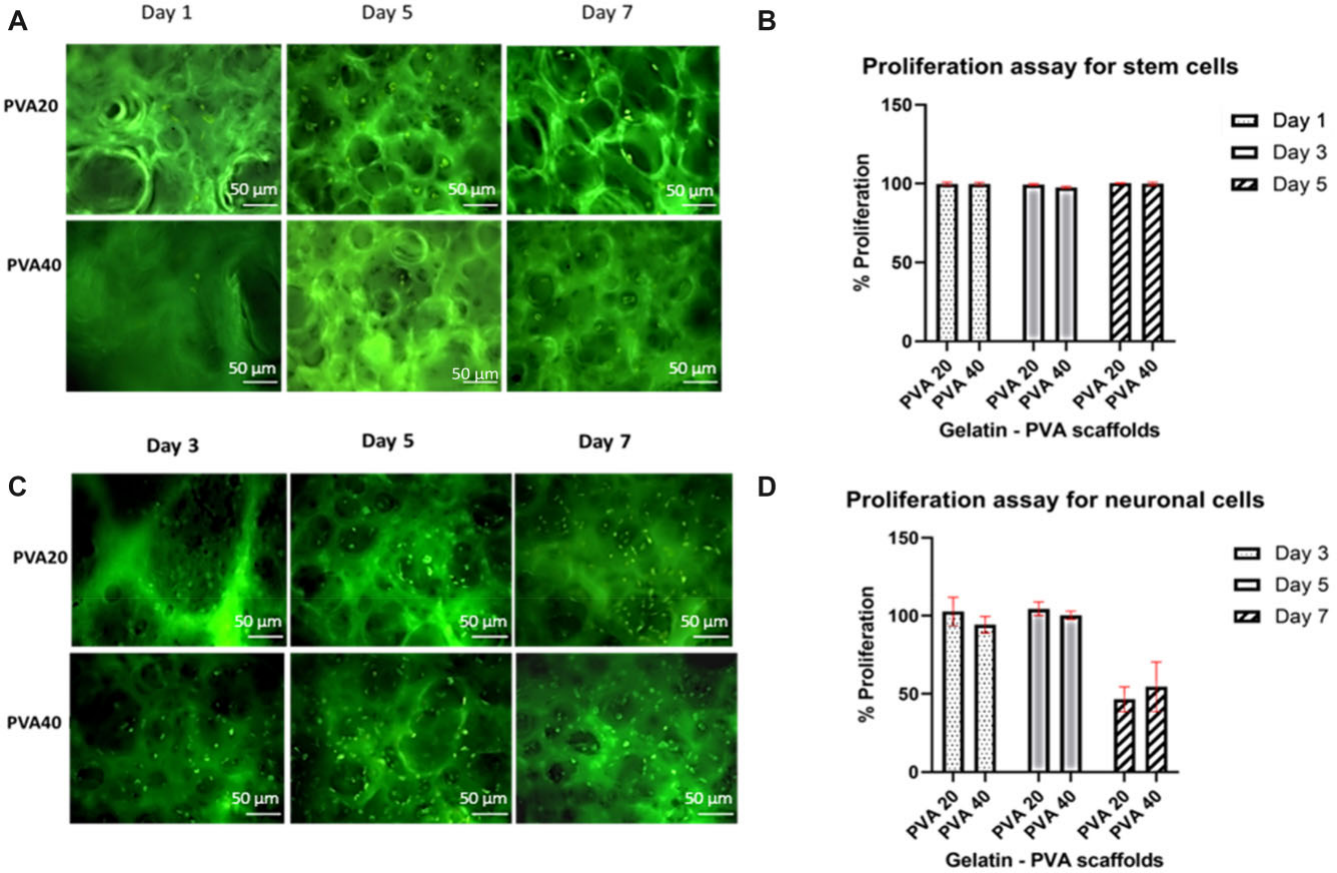
Validation of the scaffolds. The cell viability of stem cells at Day 1, 3 and 5 on PVA20 and PVA40 (×10). The penetration of the cells within the pores of the scaffolds are visible (**A**). proliferation assay (MTT) for stem cells on Day 1, 3 and 5 showing >95% viability. There is no significant difference in the proliferation pattern (**B**). The cell viability of AA differentiated neuronal cells at Day 5 and 7 on PVA20 and PVA40 (×10) (**C**). Proliferation assay (MTT) for stem cells on Day 3, 5 and 7 showing 25% decrease in the proliferation on Day 7. There is no significant difference in the proliferation pattern (**D**).

Previous reports suggest that the stem cells from early passage (primary cells) stem cells have high proliferation rate [42]. The stem cell proliferation on the scaffolds (after seeding on PVA 20 and PVA 40) were studied at Day 1, 3 and 5, using the MTT assay. This quantitative assay also emphasize that the fabricated 3D matrix is not cytotoxic to cells. In addition, the viability analysis of the primary cells on the scaffolds was evaluated using *in situ* AO and PI staining. The Passage 2 stem cells (2× 10^4^ cells) were seeded on the scaffolds uniformly. Figure 7A exhibits all live nucleated cells in fluoresce green and there were no notable dead cells which fluoresces red, indicating that the scaffold constituents were not cytotoxic. The live ASCs reduce the tetrazolium salts to their formazan crystals. Thus, the concentration of ASCs is directly proportional to the measured optical density. Figure 7B, displayed >95% of viability and growth of ASCs seeded on the scaffold was observed. The scaffolds had no significant difference in the pattern of proliferation for the stem cells on Day 1, 3 and 5. Altogether our observations revealed that the scaffolds are non-toxic and cytocompatible which support the growth and proliferation of cells.

AA has been reported to enhance differentiation of embryonic stem (ES) cells into neurons strikingly, the antioxidant epigenetically enhances midbrain dopamine neurons [43, 44]. In the current study, to test the differential potential of ASCs, the late passage ASCs (transit-amplified) was transdifferentiated to neurons using single signalling factor AA (Fig. 2). Additionally, we hypothesize that the high porous scaffold with lower mechanical strength would support soft tissue engineering especially neural lineage. The differentiated neural-lineage cells were seeded on the scaffold. The MTT assay showed that the scaffold supported the growth and proliferation till Day 5 and there is 50% reduction in the proliferation rate at Day 7 (Fig. 7C). At the same time, the AO/PI staining did not exhibit any dead cells which fluoresce red (Fig. 7D). The reduction in proliferation rate may be due to the attainment of terminal differentiation of neural-lineage cells which no longer able to divide mitotically defined as post-mitotic.

### *In situ* differentiation of stem cells to neuronal cells on the gelatin/PVA matrix proved the efficacy of the scaffold

**Figure 8.**
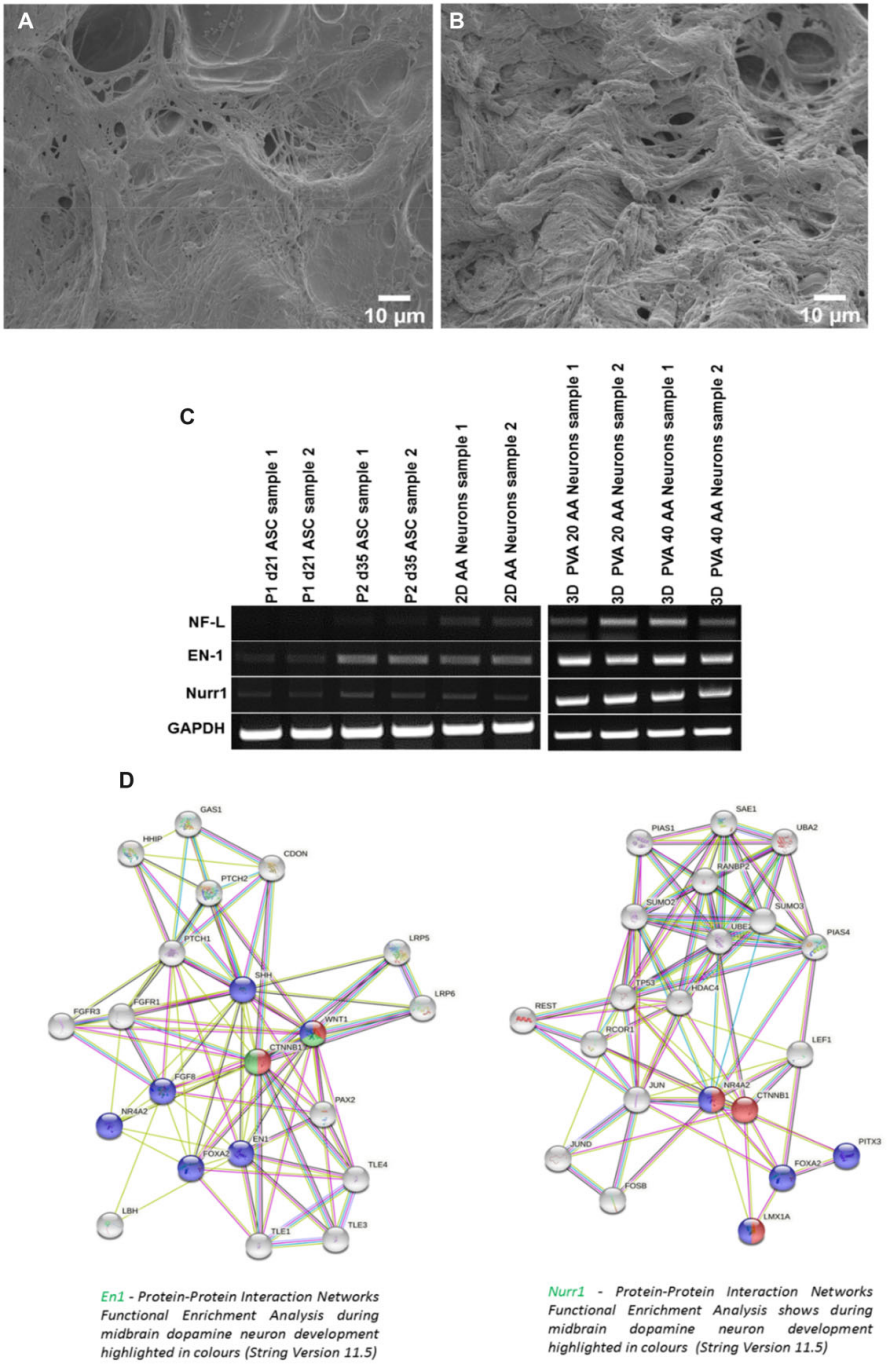
Efficacy of the scaffolds. Cell–biomaterial interaction on PVA scaffolds PVA20 (**A**) and PVA40 (**B**). The gene expression of the neuronal specific marker NF-L, NURR1 and EN1 in 2D and 3D (**C**). GAPDH serves as the endogenous control. The protein–protein interaction of NURR1 and En1 is shown in string analysis (**D**).

As later passages of ASCs readily differentiate into neurons, we have now tried to differentiate early passages of ASCs *in situ* in the scaffold to facilitate faster and effective differentiation to neural-lineage cells. To check the efficacy of the scaffold to support the differentiation of stem cells to neuronal lineage, the stem cells from Passage 2 were seeded on the scaffolds where the cells seeded on the TCPs served as the 2D control. The cells in the scaffold and plates were maintained in AA medium for a period of 21 days after which they were characterized using semi quantitative RT-PCR. The obtained results revealed that intermediate filament NF-L specific to neuronal cells was highly expressed in the 3D scaffold rather than 2D environment. The cell biomaterial interaction was studied using SEM, where the cellular adhesion and proliferation on the scaffolds were visible. The elongated cellular morphology can be visualized in Fig. 8A and B, where the capability of the material to support differentiated neuronal cells is proven. Therefore, the scaffold has been proven to be the good supporting environment for *in vitro* neuronal differentiation.

The vital role of AA (signalling molecule) in midbrain dopamine neuron development has been demonstrated in numerous studies [13–18]. But, unlike other studies the current study uses AA as single signalling molecule. Hence to confirm the neuronal differentiation of the ASCs towards dopaminergic neurons, the mesencephalic dopaminergic neuronal genes such as En1 and Nurr1 were analysed. The En1 and Nurr1 are highly expressed in 3D scaffolds compared with cells grown in TCPs, thus confirming the dopamine neuron like differentiation of ASCs (Fig. 8A). Finally, the gelatin–PVA blended matrix also proven to be transmitting the nutrients and signalling molecule efficiently.

The protein–protein interaction of En1 and Nurr1 and its correlation with other proteins involved in midbrain DA neuron development has been shown using string analysis in Fig. 8B.

## Discussion

In the present study, we attempted to design a simple and effective approach to restore soft tissues and investigate the developmental aspects with the objective to repair the damaged tissues like aorta, brain tissue, smooth muscle, etc. Principally, regenerative medicine revolves around the tissue engineering triad that comprises of stem cells for regeneration, biomaterials for ECM support and signals for growth, proliferation and differentiation. Soft tissues are complex fibrous tissues that are distinguished from hard tissues based on the water content in them [45]. These tissues face insults caused by trauma, surgery or degeneration due to ageing. They can be restored either by transplantation of cells or the implantation of a 3D matrix with cells [46–48]. Although these approaches are theoretically promising, the former (cell transplantation) have serious drawbacks like the dislodging of cells, suboptimal dispersion, donor site morbidity, immunogenic response and above all the lack of ECM and its proteins [49, 50]. In contrast, cells loaded in matrices when implanted at the site of injury/ischemic infarct would have ECM support. The cell–ECM interactions through adhesion and structural proteins, provide physical and chemical cues to the cells for fate determination [51, 52]. Generally, the scaffolds must provide the matrix stability, deliver signals for the tissue specific cellular processes, and degrade in concert with the regeneration of the tissue without the release of any toxic by-products that cause local or systemic inflammation.

Below the patella of the knee the stroma of the connective tissue supports uniformly arranged fat cells along with fibroblasts, macrophages, precursor cells and endothelial progenitors. This elastic tissue not only supports the frictionless motion of the knee but also serves as the rich source of stem cells with a varied array of cytokines [11, 53, 54]. Here we demonstrated the stemness of ASCs through the gene and protein expression specific to ESCs and MSCs. Nanog, a DNA binding transcription factor can both regulate pluripotency (by combining with OCT4 and SOX2) and allows differentiation through autorepression was found to be observed in early passage ASCs. The expression of nucleostemin, a proliferation marker revealed that the cells are proliferative. The mesenchymal markers such as CD44, CD105 and CD166 were highly expressed in ASCs. These structural and transcription factors are intricately involved in stem cell regulatory pathways.

Our group has previously reported that the ASCs from later passages spontaneously exhibit neuronal markers and the reductions of stem cell markers from Passage 3 [19]. In the present study, we hypothesized that the cells from Passage 4 were ‘transit amplified cells’, towards neural lineage. To further channelize the differentiation and neuronal specification, AA (vitamin C), an antioxidant is used as the only signalling factor. Indeed, various studies have validated that *in vitro* treatment of AA facilitates the development of dopaminergic neurons and also act as a neuromodulator which has remarkable neuroprotective effects [18, 44, 55–57]. On Day 15 after induction with AA, the transit amplifying cells crossed the precursor stage, which was evident from the complete absence of DCX, a precursor and immature neuronal marker. Likewise, the absence of transcription factors, ASCL (pro-neural gene) and OLIG2 (multipotent precursor marker) revealed the same. The predicted neural lineage was well established through the expression of transcription factors Nurr1 and En1. Nurr1 belongs to the family of ligand independent nuclear receptors activated by steroid thyroid activation factor. Nurr1 deficiency in the ventral midbrain of animals resulted in loss of all the genes or markers specific to dopaminergic neurons which include TH and dopamine transporter. In addition, Nurr1 also positively regulates the translation of TH gene by the recruitment of transcriptional co-activators. Similarly, En1 a homeobox transcription factor that primarily regulates the dorsal midbrain and mesencephalic dopamine neurons by maintaining the signalling factor Fgf8. Nurr1−/− and En1−/− mice lack functional mesencephalic dopaminergic neurons with poor axonal outgrowth that eventually leads to death. The functionality of the differentiated neurons in neurotransmitter signalling was described by the expression of D4 receptor, involved in regulating the functions of prefrontal cortex [29, 58–61]. Furthermore, the neural specific cytoskeletal protein expression such as NF-L, NF-M and TUBB-3 revealed that the AA signalling enhanced neural lineage differentiation with appropriate specificity.

Soft tissues or specialized tissues exhibit high flexibility and a very low mechanical stiffness with a maximum of 1 MPa [5], such physiological features endow the topographical, mechanical and biochemical properties of a tissue [62]. As mentioned earlier the primary goal of this study is to design a promising model for soft tissue engineering which matches the native tissue. The study was conducted using a blend of gelatin, a hydrolysed form of collagen and PVA, which exhibit tissue-like elasticity that mimic brain tissues which have a high concentration of GAGs and lower amount of collagen. A TS ranging from 0.2 MPa (PVA20 in wet condition) to 1.66 MPa (PVA40 in wet condition) has been achieved in the current study. The lower mechanical strength and elastomeric property will be very much favourable in soft tissue engineering applications like brain tissue with ∼2500 Pa/0.0025 MPa or Aorta with 0.3–0.8 MPa (7, Liu, WF 2005). The *in vivo* biocompatibilty of the synthesized scaffolds was assessed using whole blood and the enzyme lysozyme as they are first exposed to blood and body fluids when used as an implant. *Invivo* degradation of a scaffold is considered as a crucial factor. While aiding in the regeneration of the host tissue the scaffolds slowly lose their mechanical and physiochemical properties. Principally, the remaining debris of the scaffolds will be cleared by the immune cells of the host [63]. The components of the scaffold were found to be hemocompatible as there is no notable lysis of RBCs or thrombus formation (Fig. 5C and D). PVA endows resistance against degradation which slows down the overall disintegration (Fig. 5A). The cell distribution and the vascularization in the scaffold is attributed to the porosity [62, 64]. The solvent replacement method revealed that the porosity of PVA20 was 99% and the increase in gelatin concentration in PVA40 resulted in a 70% decrease in porosity. Accordingly, the 3D matrix PVA 20 is likely to support cell ingrowth and vascularization. The vascularization can also be enhanced by the inclusion of pre-vascularization factors such as VEGF, CTCF, TGF-β, PDGF or through the co-culture of endothelial cells [65, 66]. In addition, the thermal stability of the scaffold was observed at body temperature (37°C).

The cytocompatibility of the scaffolds was studied using primary stem cells isolated from IFP and differentiated neural lineage cells derived from ASCs. The colorimetric assay for measuring the cell metabolic activity (MTT) and the measurement of cell viability using AO/PI demonstrated that the scaffolds were cytocompatible (Fig. 6A and C). The fluorescent micrographs of the cells stained with AO/PI (stem cells and neural lineage cells) not only revealed the viability of the cells, but also the cell–scaffold interaction through adhesion and the migration of the cells in the multilayers of the 3D matrix. The MTT assay on Day 7 with neural cells, revealed a 50% reduction in the cell proliferation which may be due to the terminal differentiation aided by 3D matrix.

To further analyse the efficacy of the scaffold as a neural tissue construct model, the cellular behaviour in response to the physiochemical changes were studied *in situ* using gene expression analysis. The relatively high expression of the neuron-specific intermediate filament, NF-L in 3D matrix revealed that the structural integrity of the neural lineage cells was maintained inside the scaffolds. Additionally, the of En-1 and Nurr-1, the factor specific for the induction of mesencephalic dopaminergic neurons was predominantly expressed in the cells grown in 3D matrix. The gelatin–PVA blended matrix also proven to be transmitting the nutrients and AA signalling molecule efficiently. It is clear that AA plays a crucial role in Dopamine neuron specificity determination. In every way, the topography of the scaffolds supports the cell adhesion, migration, proliferation, differentiation, cytoskeletal organization, and signal transduction. To facilitate the implantation, we suggest that gelatin–PVA can be synthesized as a hydrogel with the same crosslinking agent without freeze drying.

Although the role or importance of AA in the development/differentiation of neurons has been demonstrated in various studies, to our knowledge this is the only study where AA has been used as the single signalling factor in neuronal fate determination. Wulansari *et al*. [57] have reported that AA induces the neuronal differentiation epigenetically through the upregulation of a panel of neuron specific developmental genes through the deletion of DNA methylation and repressive H3K9m3/H3K27m3 at the gene promoter regions resulting in dopaminergic neurons. These neurons express NURR1 and En1 which are essential for dopaminergic neuron survival and maintenance [67] which has also shown in the current study. Mechanistically, Bai *etal*. [18] reported that AA functions through the MEK-ERK1/2 pathway by the manipulation/remodelling of collagen synthesis where AA plays a role in cell adhesion, ECM deposition and collagen synthesis. This study also identified the transit amplified cells at Passage 4, which can be readily used for the differentiation of specified neurons. Over the past decade our group has been intensively working on IFP derived stem cells and their applications. We now strongly recommend the use of IFP derived stem cells as a novel candidate for cellular therapy and regenerative medicine. From the observations we determine that the fabricated gelatin–PVA implantable 3D matrix is an appropriate niche. The niche could be provided for *in situ* growth and differentiation of stem cells which would facilitate regeneration of structural and functional soft tissues. Even though we conducted studies to prove the fate determination and the functionality of neurons in 2D and 3D, the lack of neurotransmitter quantification and confocal z-stacking imaging are considered as technical limitations of the present study.

In conclusion, the ASCs serve as a novel candidate for tissue engineering, especially neural regeneration. Similarly, AA a potent detoxifying antioxidant serves as a single cell signalling molecule, for dopamine neuron fate determination. In the future, production of tubular scaffolds with intact tubular opening could be fabricated with gelatin–PVA blend for the support of endothelial cells in vascular tissue engineering. The successful management of soft tissue loss in vascular system ranging from microvasculature to aorta enables the enhancement of regeneration, and reduce the morbid conditions in low cost. For huge loss of tissues, the same blend and autologous ASCs could be prepared as a book type construct or lego-type construct using cell sheet engineering technology which would replace the flap construction with difficult dissection and longer operative time. Overall, we have discussed the various mechanisms by which the same blend with cells can be constructed for soft tissue engineering. As an additional effort, our team concentrates in construct for neural tissue with guiding molecules such as netrins, semaphorins and ephrins. Thus, the cost effective gelatin–PVA matrix together completes the tissue engineering triad of this present study. This combinatorial approach could emerge as a novel model for soft tissue engineering.
